# Galvanic Tongue Stimulation Inhibits Five Basic Tastes Induced by Aqueous Electrolyte Solutions

**DOI:** 10.3389/fpsyg.2017.02112

**Published:** 2017-12-05

**Authors:** Kazuma Aoyama, Kenta Sakurai, Satoru Sakurai, Makoto Mizukami, Taro Maeda, Hideyuki Ando

**Affiliations:** ^1^School of Interdisciplinary Mathematical Sciences, Meiji University, Tokyo, Japan; ^2^Graduate School of Information Science and Technology, Osaka University, Suita, Japan; ^3^Lighting Business Division Lighting Equipment Business Unit Development Group, Panasonic Corporation Eco Solutions Company, Kadoma, Japan; ^4^Microsoft Japan Co., Ltd., Tokyo, Japan; ^5^Center for Information and Neural Networks, National Institute of Information and Communications Technology, Suita, Japan

**Keywords:** taste, electrical stimulation, electrical tongue stimulation, taste inhibition, virtual taste

## Abstract

Galvanic tongue stimulation (GTS) modulates taste sensation. However, the effect of GTS is contingent on the electrode polarity in the proximity of the tongue. If an anodal electrode is attached in the proximity of the tongue, an electrical or metallic taste is elicited. On the other hand, if only cathodal electrode is attached in the proximity of the tongue, the salty taste, which is induced by electrolyte materials, is inhibited. The mechanism of this taste inhibition is not adequately understood. In this study, we aim to demonstrate that the inhibition is cause by ions, which elicit taste and which migrate from the taste sensors on the tongue by GTS. We verified the inhibitory effect of GTS on all five basic tastes induced by electrolyte materials. This technology is effective for virtual reality systems and interfaces to support dietary restrictions. Our findings demonstrate that cathodal-GTS inhibits all the five basic tastes. The results also support our hypothesis that the effects of cathodal-GTS are caused by migrating tasting ions in the mouth.

## Introduction

Galvanic tongue stimulation (GTS) induces an electric or metallic taste (Stevens et al., 2008). This fact was first discovered by Sulzer in the 18th century. The technology has been used for gustatory testing in medical research and as a tool in neural science (Krarup, 1958; Von Békésy, 1964; Cardello, 1981). As GTS can induce taste without consumption of solids or liquids, this technology would be effective in providing a virtual consumption experience and in supporting dietary restrictions. Therefore, GTS is likely to be employed for virtual reality and health engineering purposes (Vazquez-Buenosaires et al., 2003; Aruga and Koike, 2015; Ranasinghe and Do, 2017). For such engineering applications, GTS should be able to preferably regulate all the five taste sensations, i.e., sweetness, bitterness, saltiness, sourness, and umami. However, the electrical taste induced by GTS is extraordinarily complex and it has been generally known as a method to induce electrical taste (Lawless et al., 2005). Conventional studies about GTS reported that an electrical taste is induced when the anode is in the proximity of the tongue (anodal-GTS) (Platttig and Innitzer, 1976; Ranasinghe et al., 2013). On the contrary, Hettinger and Frank (2009) demonstrated that GTS inhibits the taste of salt solutions when the cathodal electrode alone is placed in the subject’s mouth (cathodal-GTS). Although their studies demonstrate the inhibitory effects of cathodal-GTS on salty and bitter-salty tastes, the group did not investigate whether other basic tastes were affected. Moreover, the inhibitory mechanism was not adequately investigated.

**FIGURE 1:**
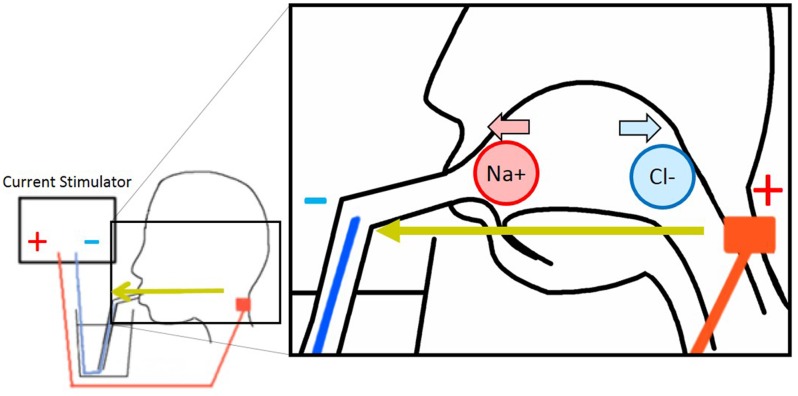
Electrode arrangement of galvanic tongue stimulation (GTS) and image of ionic migration theory.

Currently, two tentative hypothesis of the cathodal-GTS inhibitory mechanism exist: the ionic migration hypothesis and the nervous stimulation hypothesis. The ionic migration hypothesis postulates that the electrical field formed by the GTS current migrates and removes the tasting ions from the tongue (Hettinger and Frank, 2009) (**Figure 1**). In contrast, the nervous stimulation hypothesis, which is the simplest considerable alternate hypothesis, states that the electrical current disrupts nerve activation or habituates nerves. In anodal-GTS, Volta and Bujas proposed that stimulation current directly affects (may depolarize) taste cells or taste nerves (Bujas, 1971; Nakamura and Miyashita, 2016). Therefore, it is rational to consider that in cathodal-GTS, the cathodal current directly deactivates nerves or brings a negative effect. The objective of the present study is to determine the inhibitory mechanism of cathodal-GTS, and to investigate whether it influences all five basic tastes.

The gustatory sensors, referred to as the taste buds, detect chemicals in the mouth, which constitute the sense of taste. Each taste bud contains taste cells, which have receptors at the surface of the cell (Chandrashekar et al., 2006) that detect materials in aqueous solutions (Kandel et al., 2014). From this perspective, materials that generate tastes can be divided into two categories: electrolytes and non-electrolytes. If the nervous stimulation hypothesis is valid, the cathodal-GTS should inhibit the taste induced by both electrolytes and non-electrolytes. However, if the ionic migration hypothesis is valid, then the cathodal-GTS should inhibit only the taste induced by electrolytes. As it has not been demonstrated that cathodal-GTS inhibits tastes apart from saltiness, we support the ionic migration hypothesis as the mechanism of taste inhibition by cathodal-GTS.

To demonstrate the validity of the ionic migration hypothesis, we conducted an experiment in which subjects were exposed to four types of aqueous solutions, i.e., sucrose, glycine, caffeine, and MgCl_2_. Sucrose and glycine exhibit sweet tastes, and caffeine and MgCl_2_ exhibit bitter tastes. Sucrose and caffeine are non-electrolytes, while glycine and MgCl_2_ are electrolytes (Stone and Oliver, 1969). We used only those materials that exhibit sweetness and bitterness because non-electrolytes that elicit umami, acidity, or salty tastes are unknown. For this experiment, subjects regulated concentrations of sucrose and MgCl_2_ solutions so that the strengths of the two become equivalent to the strength of glycine and caffeine solutions, respectively. The effect of cathodal-GTS on the subject’s capability to taste electrolyte and non-electrolyte solutions was investigated using these fixed concentrations. To analyze their capability to taste sweetness, subjects tested the following pairs: glycine with GTS vs. without GTS, sucrose with GTS vs. without GTS, glycine without GTS vs. sucrose without GTS, and glycine with GTS vs. sucrose with GTS. To analyze their capability to taste bitterness, subjects tested the following pairs: MgCl_2_ with GTS vs. without GTS, caffeine with GTS vs. without GTS, MgCl_2_ without GTS vs. caffeine without GTS, and MgCl_2_ with GTS vs. caffeine with GTS. Subjects verbally answered whether one of the samples had a stronger taste than the other or not.

Subsequently, we investigated whether cathodal-GTS could inhibit all five basic tastes induced by aqueous electrolyte water solutions, and the relation between the strength of cathodal-GTS and the inhibitory effect on taste. Subjects were provided with five types of aqueous electrolyte solutions, namely, NaCl, MgCl_2_, glycine, glutamic sodium, and citric acid, with salty, bitter, sweet, umami, and sour tastes, respectively. Each type of solution was prepared in five concentrations (**Table 1**). The solution with the highest concentration in each type was termed the “adjusting sample,” while the others were termed “comparable samples.” In each trial, a subject first tasted the comparable sample. Then, the subject attempted to adjust the taste intensity of the adjusting sample to match it to that of the comparable sample by altering the current intensity of the cathodal-GTS.

**Table 1:** Consistency of adjusting material and comparable materials in the second experiment.

	Adjusting material [%]	Comparable materials [%]			
Glycine	5.0	4.0	3.0	2.0	1.0
MgCl_2_	0.50	0.40	0.30	0.20	0.10
NaCl	1.0	0.80	0.60	0.40	0.20
Glutamic sodium	0.50	0.40	0.30	0.20	0.10
Critic acid	0.50	0.40	0.30	0.20	0.10


Therefore, the contributions of this work to the field of electrical taste, taste physiology, or taste psychology are the following:

(1) to reveal the mechanisms of taste inhibitory effect of cathodal-GTS, and
(2) to demonstrate that cathodal-GTS inhibits taste induced by electrolyte materials regardless of taste quality.

## Materials and Methods

All the experiments complied with the safety standards approved by the local ethics research committee at the Graduate School of Information Science and Technology, Osaka University, Japan. All the participants had the experiments explained to them and signed a letter of consent. The study protocol was performed in accordance with the ethical standards laid down in the Declaration of Helsinki.

In terms of safety thresholds for electrical stimulation, we followed the safety guidelines for transcranial direct current stimulation (tDCS). Since there are no standard limitations for GTS, various stimulation strengths and durations were used in previous studies, e.g., up to approximately 55 mA but some studies described the voltage instead of the current strength (Aruga and Koike, 2015; Khan et al., 2016). tDCS is a technique for modulating brain and neuronal excitability by stimulating the head electrically. The paper published by Bikson et al. (2016) indicated that the use of conventional tDCS protocols in human trials for up to 40 min and at 4 mA has not produced any reports of serious adverse effects or irreversible injury in over 1000 subjects. GTS is similar to tDCS in that it stimulates the head electrically. Therefore, we believe that the stimulation limitations for tDCS should be employed for GTS. In this work, we employed safer limitations than that of tDCS, i.e., the stimulation current is only up to 2.5 mA and the longest stimulation duration is 30 s to ensure the safety of subjects. In fact, all subjects used up to 1 mA of current except for one subject who used a maximum current of approximately 2.3 mA.

In all experiments, we used an in-house constant current stimulator, which consists of an operation amplifier (LMC6482), MOSFET (2SK3113), and transistor (2SA1413). This circuit is driven by a microcontroller (18F2620) that receives commands from a PC.

### Effect of Cathodal-GTS on Sweet and Bitter Tasting Electrolyte and Non-electrolytes

Eight male participants enrolled in this experiment. An anodal electrode (NIPLODE, Fukudadenshi, Inc., Tokyo, Japan) was attached to the back of the neck, and a conductive wire that passed through a drinking straw was used for the cathode (**Figure 1**). The reason why we attached the anodal electrodes to the back of the neck is because this position induces white flash visual sensations and ticking sensations on the skin vanishingly compared with electrode positions in previous works (Hettinger and Frank, 2009; Aruga and Koike, 2015). During the preliminary phase of this experiment, subjects adjusted the concentrations of sucrose and MgCl_2_ aqueous solutions so that they exhibit taste strengths identical to those of aqueous solutions of 5.0% glycine and 0.3% caffeine, respectively. Using these fixed sucrose and MgCl_2_ samples and the 5.0% glycine and 0.3% caffeine samples, subjects were presented four types of conditional sample-pairs for each taste quality. The following pairs were presented for the sensation of sweetness: glycine with GTS vs. without GTS, sucrose with GTS vs. without GTS, glycine without GTS vs. sucrose without GTS, and glycine with GTS vs. sucrose with GTS. The following were presented for the sensation of bitterness: MgCl_2_ with GTS vs. without GTS, caffeine with GTS vs. without GTS, MgCl_2_ without GTS vs. caffeine without GTS, and MgCl_2_ with GTS vs. caffeine with GTS. Cathodal-GTS was applied using a 1.0-mA and 2000-ms square current. In each trial, the subject tasted each of the two comparable solutions once. Each condition was repeated four times, and the 16 trials were conducted in a random sequence for each taste quality.

### Relation between the Strength of Cathodal-GTS and Inhibitory Influences on the Five Basic Tastes

Six adult male participants enrolled in this experiment. An anodal electrode (NIPLODE, Fukudadenshi, Inc., Tokyo, Japan) was attached to the back of the neck, and a conductive wire that passed through a drinking straw was used as the cathode. Solutions of glycine, MgCl_2_, NaCl, glutamic sodium, and citric acid were used as the taste samples.

Each solution was prepared in five concentrations (**Table 1**); the solution with the highest concentration was termed “adjusting sample,” while the others were termed “comparable samples.” In each trial, a subject first tasted the comparable sample. Then, the subject was asked to adjust the strength of the taste of the “adjusting sample” to that of the “comparable sample” by using a sliding volume controller connected to the stimulator. This sliding volume was connected to a microcontroller (NXP mbed LPC 1768) which sends commands to a PC, which then sends commands to the constant current stimulator. The current strength ranged from 0 to 2.5 mA and longest current duration was 30 s. However, all subjects finished adjusting the slide volume within 30 s. Each combination of the adjusting and comparable samples for each taste quality was tested six times. Thus, each subject completed 120 trials. Owing to safety limitations, the trials for only one tasting sample were conducted in 1 day. The sequence of the trials was randomized. The sampled current intensity data were normalized for each subject by dividing the data by the maximum current intensity recorded for the subject.

The differences in conditions between the first and second experiments are shown in **Table 2**.

**Table 2:** Experimental conditions.

	Experiment 1	Experiment 2
Stimulation strength [mA]	1.0	0–2.5
Stimulation duration [ms]	2000	0–30000
Waveform	Square	Manual
Answer	Verbal	Adjust current
Number of trials [times per taste quality]	12	24


## Results

### Examining the Influence of Cathodal-GTS on Sweet and Bitter Tasting Electrolyte and Non-electrolytes

**FIGURE 2:**
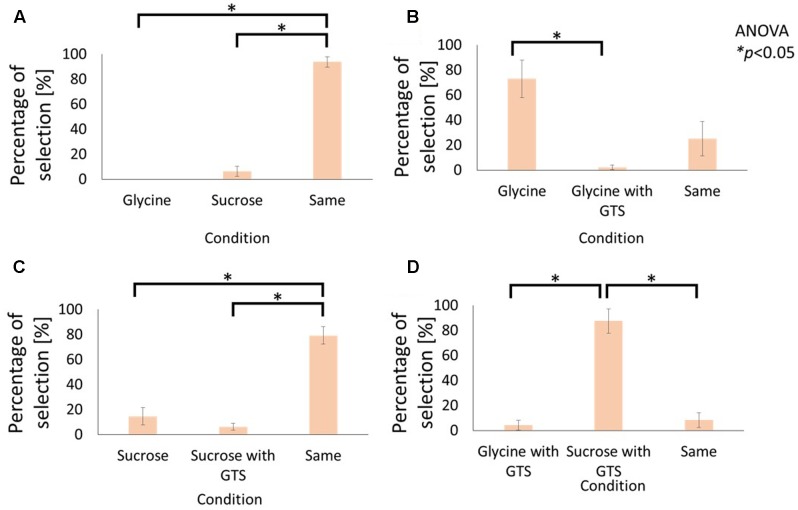
Influence of cathodal-GTS on sweetness of electrolyte and non-electrolyte aqueous solutions. These figures indicate the percentage of selection in the following conditions: **(A)** Glycine vs. Sucrose without GTS, **(B)** Glycine without GTS vs. with GTS, **(C)** Sucrose without GTS vs. with GTS, **(D)** Glycine with GTS vs. Sucrose with GTS. Data are reported as mean ± standard error of mean. Asterisks (^∗^) in these figures reveal the significant differences (*p* < 0.05) detected by statistical analysis using Kruskal–Wallis ANOVA and multiple comparisons test (Scheffe’s method).

**FIGURE 3:**
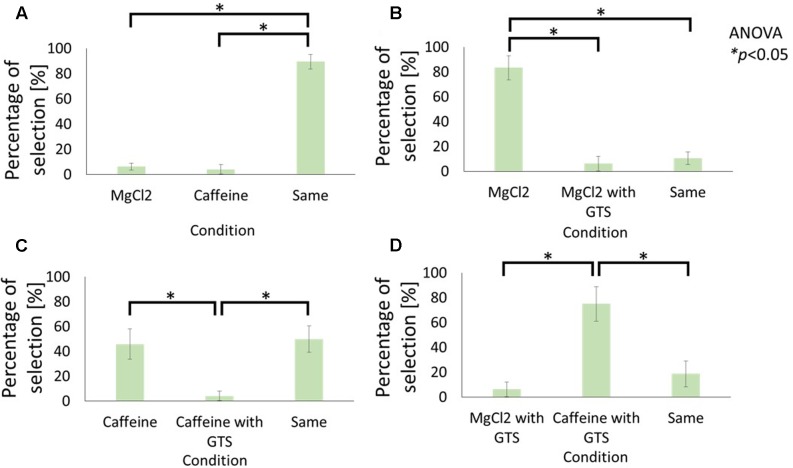
Influence of cathodal-GTS on the bitterness of electrolyte and non-electrolyte aqueous solutions. These figures indicate the percentage of selection in each condition: **(A)** Caffeine vs. MgCl_2_ without GTS, **(B)** MgCl_2_ without GTS vs. with GTS, **(C)** Caffeine without GTS vs. with GTS, **(D)** Caffeine with GTS vs. MgCl_2_ with GTS. Data reported as mean ± standard error of mean. Asterisks (^∗^) in these figures reveal the significant differences (*p* < 0.05) detected by statistical analysis using Kruskal–Wallis ANOVA and multiple comparisons test (Scheffe’s method).

**Figures 2** and **3** illustrate the results for the sensations of sweet and bitter tastes, respectively.

**Figures 2A** and **3A** compare the average selection percentage between the electrolytes and non-electrolytes in eliciting a sensation of sweetness and bitterness, respectively. Statistical analyses, performed with Kruskal–Wallis analysis of variance (ANOVA) and multiple comparisons (Scheffe’s method) test, on the response percentages demonstrated that the percentage of “similar level” responses were significantly higher than the others (**Figure 2A**: F2, 21 = 8.55, *p* < 0.05; **Figure 3A**: F2, 21 = 11.81, *p* < 0.05). This indicates that the strength of taste of the electrolyte and non-electrolyte samples were sensed as being similar.

**Figures 2B** and **3B** demonstrate the effect of cathodal-GTS on the sensations of sweetness and bitterness, respectively, which were elicited by the electrolytes. Statistical analyses performed using the Kruskal–Wallis ANOVA and Scheffe’s method discovered that Cathodal-GTS weakened both the taste sensations (**Figure 2B**: F2, 21 = 16.28, *p* < 0.05: **Figure 3B**: F2, 21 = 18.01, *p* < 0.05). However, cathodal-GTS did not demonstrate an effect on the sweet and bitter tastes that were elicited by the non-electrolytes, i.e., sucrose and caffeine, as the percentages of “similar level” responses were significantly higher in comparison with the other conditions (**Figure 2C**: F2, 21 = 19.92, *p* < 0.05; **Figure 3C**: F2, 21 = 9.51, *p* < 0.05).

Finally, the effect of cathodal-GTS on the electrolyte and non-electrolyte, eliciting sweetness or bitterness, are illustrated in **Figures 2D** and **3D**, respectively. Cathodal-GTS affected only the taste sensation of the electrolytes, i.e., glycine and MgCl_2_ (**Figure 2D**: F2, 21 = 17.28, *p* < 0.05; **Figure 3D**: F2, 21 = 16.15, *p* < 0.05).

### Investigating the Relation between the Strength of Cathodal-GTS and Inhibitory Effects on the Five Basic Tastes

**FIGURE 4:**
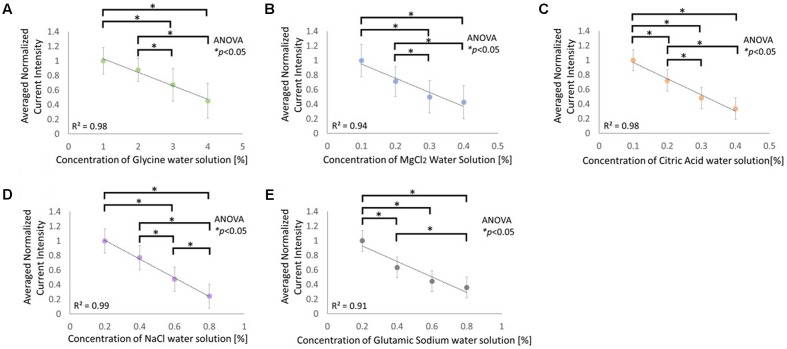
Relationship between current intensity required to match the taste intensity of the adjusting sample to the comparable sample of the following solutions: **(A)** glycine, **(B)** MgCl_2_, **(C)** citric acid, **(D)** NaCl, and **(E)** glutamic sodium. Asterisks (^∗^) in these figures reveal the significant differences (*p* < 0.05) detected by statistical analysis using Kruskal–Wallis ANOVA and multiple comparisons test (Scheffe’s method).

**Figure 4** illustrates the normalized and averaged current strength that was required to inhibit the taste of the adjusting sample so that the taste strength was similar to the strength of comparable sample. The solutions used were glycine, MgCl_2_, citric acid, NaCl, and glutamic sodium, which elicit sweet, bitter, sour, salty, and umami tastes, respectively. The concentration of the comparable sample was negatively correlated with the strength of the current for all the five types of electrolyte solutions. Statistical analyses performed using the Kruskal–Wallis ANOVA and Scheffe’s method for each graph demonstrated significant variations between the conditions (**Figure 4A**: F3, 140 = 72.54, *p* < 0.05; **Figure 4B**: F3, 140 = 72.82, *p* < 0.05; **Figure 4B**: F3, 140 = 81.47, *p* < 0.05; **Figure 4B**: F3, 140 = 98.42, *p* < 0.05; **Figure 4B**: F3, 140 = 75.52, *p* < 0.05).

## Discussion

The results of the first experiment demonstrate that, while cathodal-GTS does not effectively inhibit tastes induced by non-electrolyte solutions, it inhibits those induced by electrolyte solutions. **Figures 2A** and **3A** indicate that the electrolytes and non-electrolytes elicited tastes with similar strengths. However, when cathodal-GTS was applied, it weakened the sensation of the taste elicited by the electrolytes: glycine and MgCl_2_ (**Figures 2B**, **3B**); however, it did not adequately influence the non-electrolytes: sucrose and caffeine (**Figures 2C**, **3C**). The fact is further emphasized by the results illustrated in **Figures 2D** and **3D**.

Cathodal-GTS inhibited the sensation of sweet and bitter tastes elicited by the electrolytes, but did not effectively for non-electrolytes. These results support the ionic migration hypothesis as the mechanism of the taste inhibition. The major dissimilarity between electrolyte and non-electrolyte aqueous solutions is the production of ions. Electrolytes become ions in solution, while non-electrolytes do not. Therefore, ionic migration occurs when a current stimulation is applied to the electrolyte solution. During ionic migration, the electrical field formed by the GTS current impels cations and anions toward the cathode and anode, respectively. The ionic migration hypothesis matches with our experimental results that only the tastes induced by the electrolyte solutions were inhibited by the electrical field.

**Figure 4** indicates that the five basic tastes induced by electrolytes were inhibited by cathodal-GTS, and that this effect is strongly correlated with the strength of the current. The correlation coefficient for each condition was 0.9 or higher. Therefore, the strength of taste sensation is highly likely to be regulated by the strength of the current.

All subjects reported that the taste strength they experienced immediately after the stimulation offset was stronger than that they experienced prior to stimulation for all the electrolyte solutions. Hettinger and Frank (2009) reported a similar phenomenon with salty and bitter-salty solutions. However, in the first experiment in our study, the subjects did not experience taste enhancement with the non-electrolyte solutions. This indicates that the taste enhancement is caused by the preceding taste inhibitory effect. We have two descriptions of the mechanism of taste enhancement. The first is the nerve adaptation hypothesis, which postulates that taste receptors and nerves adapt to the low taste-intensity environment while GTS was applied, and then, is more strongly activated by a normal taste-intensity when GTS was stopped, resulting in the taste enhancement. Another description is that the taste ions that were attracted to each pole became diffuse when GTS ceased. The taste receptors on the tongue would be stimulated at a high frequency, resulting in a temporarily stronger taste sensation. The current study, however, is not sufficient to reveal which description is appropriate.

During the first experiment, one subject for MgCl_2_ and two subjects for glycine reported that cathodal-GTS did not inhibit taste. However, they reported the inhibition with a similar configuration on another day. On the other hand, those who continuously reported the effect reported that the strengths of the effects differ day to day. We recognize that these occurrences are results of alterations in the physical conditions of the subjects and the precise positions of the electrodes and the subjects’ tongues. Especially, since glycine is zwitter ion, the state of the inner mouth (e.g., pH and so on) may hugely affect the state of ions of glycine (Tse et al., 1978).

Moreover, some subjects verbally reported that during trials for citric acid, they felt electrifying sensations on their tongues and, at the end of the sessions, they had difficulty distinguishing between the effects of GTS and the masking effect caused by the electrifying sensation. Some also reported that taste was inhibited by the ticking sensation induced by the combination of citric acid and GTS.

This study validated the hypothesis that ionic migration is the mechanism of cathodal-GTS, which inhibits all five basic tastes. Our observations contribute to the physiological understanding of how cathodal-GTS functions, and could be applied as tools for dietary restrictions by altering meal experience.

In our preparatory experiment, we confirmed that cathodal-GTS does not affect the taste elicited by Na-saccharin. The fact is also reported in the previous works conducted by Hettinger and Frank (2009). They reported the taste inhibition for saltiness but they did not consider the state of electrolytic dissociation in the mouth. For use of cathodal-GTS in VR or diet restriction, it is preferable to control all five basic taste in the meal. To achieve this, the migration of all taste materials should be controlled. It would be our future work to model the ionic migration in the mouth, which considers degree of electrolytic dissociation, molar weight, and ionic polar character.

## Conclusion

In this work, we demonstrated that the ionic migration hypothesis is valid explanation of mechanisms for the taste inhibitory by cathodal-GTS. We also revealed that cathodal-GTS is able to inhibit all five basic tastes which is elicited by electrolyte water solutions.

## Author Contributions

KA and SS proposed the hypothesis and conducted the experiments. KA, SS, and HA designed the experiments. KS, MM, and TM assisted with the experimental setup. SS and KA collected and analyzed the data. KA, MM, and HA wrote the manuscript. All the authors discussed the results and commented on the manuscript. The works by MM and SS have done at Osaka University when they were students and are not to represent interests of Microsoft and Panasonic Corporation Eco Solutions Company.

## Conflict of Interest Statement

The authors declare that the research was conducted in the absence of any commercial or financial relationships that could be construed as a potential conflict of interest.
